# Neuritin accelerates Schwann cell dedifferentiation via PI3K/Akt/mTOR signalling pathway during Wallerian degeneration

**DOI:** 10.1111/jcmm.70012

**Published:** 2024-08-26

**Authors:** Jingmin Liu, Xin Guan, Shuai Zheng, Jiawei Shi, Xiaobo Wang, Zetao Shen, Zefu Chen, Congrui Liao, Zhongmin Zhang

**Affiliations:** ^1^ Department of Spine Orthopedics, Nanfang Hospital Southern Medical University Guangzhou China; ^2^ Department of Endoscopy Sun Yat‐sen University Cancer Center Guangzhou China

**Keywords:** dedifferentiation, demyelination, mTOR, Neuritin, Schwann cells, Wallerian degeneration

## Abstract

Neuritin, also known as candidate plasticity gene 15 (CPG15), was first identified as one of the activity‐dependent gene products in the brain. Previous studies have been reported that Neuritin induces neuritogenesis, neurite arborization, neurite outgrowth and synapse formation, which are involved in the development and functions of the central nervous system. However, the role of Neuritin in peripheral nerve injury is still unknown. Given the importance and necessity of Schwann cell dedifferentiation response to peripheral nerve injury, we aim to investigate the molecular mechanism of Neuritin steering Schwann cell dedifferentiation during Wallerian degeneration (WD) in injured peripheral nerve. Herein, using the explants of sciatic nerve, an ex vivo model of nerve degeneration, we provided evidences indicating that Neuritin vividly accelerates Schwann cell dedifferentiation. Moreover, we found that Neuritin promotes Schwann cell demyelination as well as axonal degeneration, phagocytosis, secretion capacity. In summary, we first described Neuritin acts as a positive regulator for Schwann cell dedifferentiation and WD after peripheral nerve injury.

## 1 INTRODUCTION

Neuritin, originally identified in rats and called cpg15 and subsequently in humans, is present in the central nervous system, involving neurite outgrowth and maintenance of central nervous system development.^1^, ^2^ Recent studies have shown that it is also expressed in dorsal root ganglia (DRG), involving axonal regeneration,^3^ and it is deficient in DRG neurons and axons in experimental diabetic neuropathy.^4^, ^5^ And prior evidence revealed that Neuritin acts as an important effector of androgen in enhancing peripheral nerve regeneration following injury. However, the biological functions of Neuritin in Schwann cell dedifferentiation potential during Wallerian degeneration (WD) of injured peripheral nerve have not been investigated.

WD is a progressive process of demyelination and anterograde disintegration of the distal axonal segment, and the debris of degraded axons and myelin have to be removed to provide a permissive microenvironment for the subsequent nerve regeneration.^6^, ^7^ During WD, Schwann cells are undergoing a process of dedifferentiation and demyelination, while the dedifferentiated Schwann cells plays a vital role in the clearance of the debris of axons and myelin collaborating with macrophages. Other than that, secretion capacity of dedifferentiated Schwann cells is also crucial for subsequent nerve regeneration. In the present study, we first identified the role of Neuritin in Schwann cell dedifferentiation, demyelination, phagocytosis, secretion capacity during WD after peripheral nerve injury.

## 2 MATERIALS AND METHODS

### 2.1 Experimental animals and ethics statement

Specific pathogen‐free Sprague–Dawley male adult rats (aged 8 weeks) weighing 200–250 g and neonatal rats (postnatal Day 3) were provided by the Animal Center of Southern Medical University, China (licence No. SCXK (Yue) 2016‐0041). The rats were housed in an animal room maintained at 21°C and 55% relative humidity with a 12‐h light/dark cycle and given free access to water and food. All procedures, including surgery and tissue collection, were carried out with the approval of the Southern Medical University Animal Care and Use Committee (approval No. SMU‐L2015081, approval date 15 October 2015) in accordance with the guidelines for the ethical treatments of animals. All efforts were made to minimize the number of animals used and their suffering.

### 2.2 Sciatic nerve explant culturing and treatment

Sciatic nerve explant culturing, which is an in vitro model of WD, was performed as described previously.^8^, ^9^ Briefly, rats were anaesthetised by intraperitoneal injection of 12 mg/mL tribromoethanol (180 mg/kg; Sigma‐Aldrich, St. Louis, MO, USA) and decapitated by guillotine. Sciatic nerve segments (0.5 cm in length) were isolated and incubated in the Dulbecco's modified Eagle's medium/F12 (Corning, New York, NY, USA) containing 3% fetal bovine serum (Corning), 3 mM forskolin (Sigma‐Aldrich), 10 ng/mL heregulin (PeproTech, Rocky Hill, NJ, USA), and 100 mg/mL penicillin–streptomycin (Gibco, Grand Island, NY, USA). According to the previous reports, 200 ng/mL recombinant Neuritin (PeproTech, Rocky Hill, NJ, USA) was added to the culture medium, and the nerve explants were collected after culturing for 5 or 8 days post injury (dpi) for subsequent analysis.^10^, ^11^ A vehicle‐only control group (Con group, medium without neuritin) was included.

### 2.3 Immunohistochemistry of cryosections and teased nerve fibres

Nerve segments were collected and fixed in 4% paraformaldehyde for 24 h and dehydrated in 30% sucrose overnight. Then they were embedded in optimal cutting temperature compound (Sakura Finetek, Torrance CA, USA) for cryosectioning and subsequent immunofluorescence staining. To assess the myelin ovoid formation that occurs during WD, nerve explants were also teased into individual fibres and mounted on adhesion slides.^12^, ^13^ The 10 μm‐thick cryosection slices and teased fibres were stained for immunohistochemistry as follows. The samples were first permeabilized with 0.5% Triton X‐100 (Sigma) for 30 min, then blocked with 5% fish gelatin (Sigma) containing 0.3% Triton X‐100 at room temperature for 1 h, followed by incubation with the primary antibodies diluted in blocking buffer overnight at 4°C. Goat anti‐rabbit Alexa Fluor 488 (1:400), goat anti‐rabbit Alexa Fluor 568 (1:400), and goat anti‐mouse Alexa Fluor 568 (1:400) fluorescent‐conjugated secondary antibodies (all from Molecular Probes) were applied for 2 h at room temperature. The samples were then incubated with 4′,6‐diamidino‐2‐phenylindole (1:5000; Sigma) for 2 min to counterstain the cell nuclei. Finally, images were captured using a fluorescence microscope (Leica). The primary antibodies used for immunocytochemistry were as follows: mouse anti‐myelin basic protein (MBP; 1:200, Calbiochem), rabbit antineurofilament 200 (NF200; 1:400, Sigma), mouse anti‐neuritin (1:200, Abcam), rabbit anti‐myelin‐associated glycoprotein (MAG; 1:400, Abcam), mouse anti‐c‐Jun (1:400, BD Biosciences), mouse anti‐S100 (1:100, Millipore), phalloidin (Sigma‐Aldrich).

### 2.4 Oil red O staining

To assess myelin degradation during WD, oil red O (ORO) staining was performed on the nerve explant cryosection.^14^, ^15^ Briefly, the 0.3% ORO staining solution was prepared by mixing ORO (dissolved in 60% 2‐propanol) with deionized water (ratio = 3:2). The sections were rinsed in 0.01 M phosphate buffered saline and 60% isopropanol, then incubated in the ORO solution for 15 min at 37°C and rinsed in 60% isopropanol and 0.01 M phosphate buffered saline and mounted. Images of each section were captured using a fluorescence microscope.

### 2.5 Western blot assay

Protein extracts from the collected nerve segments, as well as the in vitro cultured nerve explants, were prepared by routine procedures, then separated on 10% dodecyl sulfate sodium salt‐polyacrylamide gel electrophoresis gels and transferred to polyvinylidene fluoride membranes (BioRad, Hercules, CA, USA). After blocking with 5% bovine serum albumin (GBCBIO, Guangzhou, China) in Tris‐buffered solution containing 0.5% Tween‐20 for 2 h, the blots were probed overnight at 4°C with the following primary antibodies: rabbit anti‐glyceraldehyde 3‐phosphate dehydrogenase (1:3000, Cwbiotech), mouse anti‐MBP (1:200, Calbiochem), rabbit anti‐NF200 (1:1000, Sigma), rabbit anti‐MAG (1:400, Abcam), mouse antic‐Jun (1:400, BD Biosciences), mouse anti‐neuritin (1:200, Abcam), mouse anti‐S100 (1:100, Millipore), rebbit anti‐PI3K (1:600, Abcam), rabbit anti‐phospho AKT (1:1000, Abcam), rabbit anti‐AKT (1:1000, Abcam), rabbit anti‐mTOR (1:1000, Abcam), phospho‐mTOR (1:1000, Abcam). Next, the blots were incubated with horseradish peroxidase‐conjugated anti‐rabbit secondary antibodies (1:2000, Molecular Probes) for 2 h at room temperature, and were visualized using enhanced chemiluminescence (EpiZyme, Shanghai, China). Glyceraldehyde 3‐phosphate dehydrogenase (GAPDH) was used as an internal reference. Finally, the density was calculated using Image‐Pro Plus 6.0 software (Media Cybernetics).The relative protein expression levels were normalized to GAPDH.

### 2.6 Statistical analysis

All data are presented as mean ± standard error of the mean (SEM). All statistical analyses were performed with GraphPad Prism 5.0 software (GraphPad Software, San Diego, CA, USA) using one‐way analysis of variance followed by Bonferroni's multiple test. A *p* < 0.05 was considered statistically significant.

## 3 RESULTS

### 3.1 Temporal and spatial expression of Neuritin in ex vivo sciatic nerve explants

**FIGURE 1:**
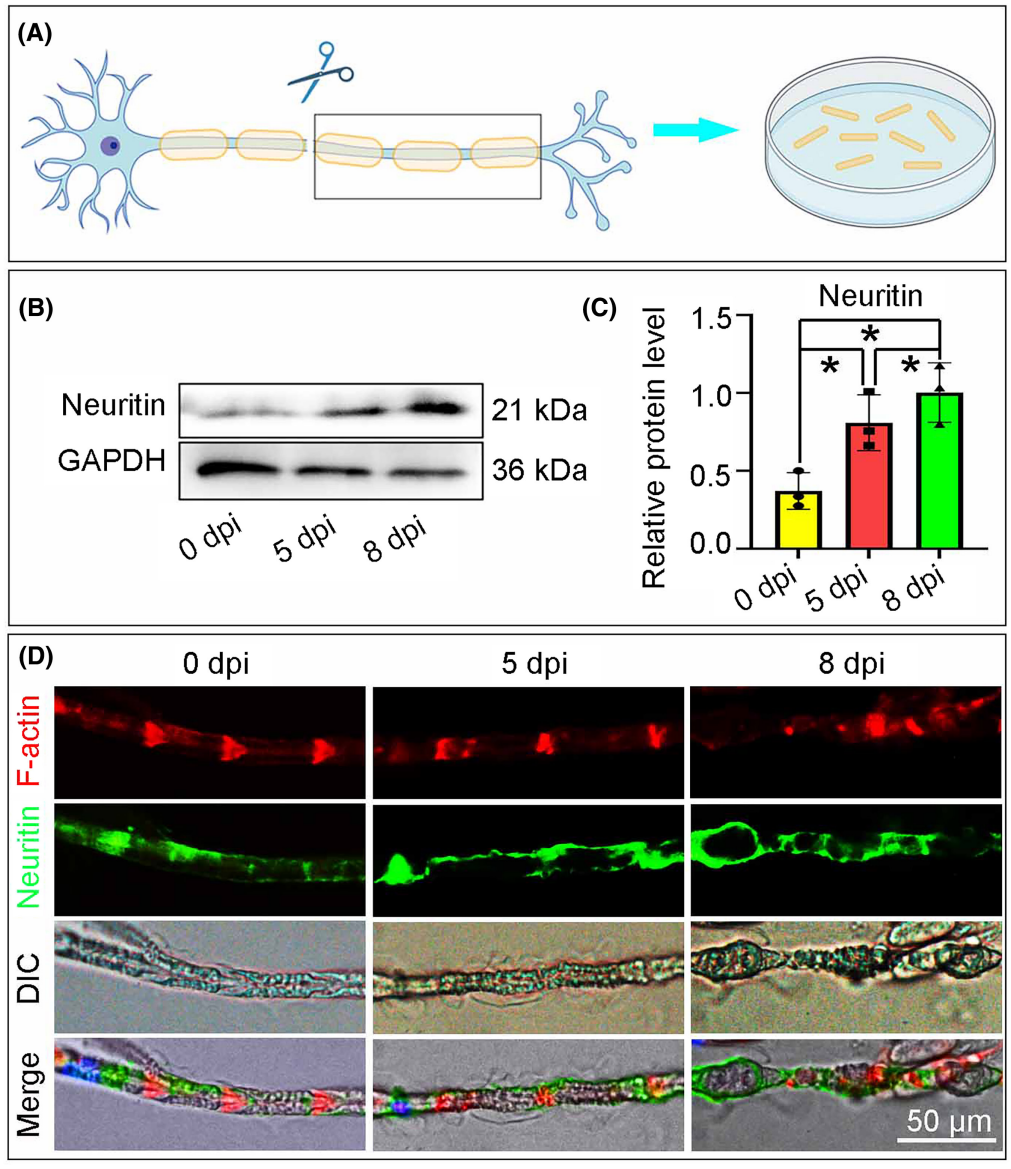
Temporal and spatial expression of Neuritin in sciatic nerve explants. (A) Establishment of ex vivo nerve explants, the sciatic nerves from 8 W rats were equant cut into segments with 3 mm length and cultured in Schwann cells medium. (B) Western blot of proteins from injured sciatic nerve at 5 and 8 dpi probed with antibodies against Neuritin. (C) Quantifcation of Neuritin protein levels in the blots. (D) Representative images showing that total Neuritin is upregulated increasingly during 5–8 dpi. And Neuritin is potently enriched in the perinuclear cytoplasm and diffused in inner mesaxon, outer mesaxon and Schmidt‐Lanterman incisure (*n* = 3, *p < 0.05).

The sciatic nerve explants were cultured in vitro for 5 or 8 days (Figure 1A). Aiming to understand the functional role of Neuritin during WD in ex vivo nerve explants, we firstly assessed the expression of total Neuritin by western blotting. Results showed that a significant increase of protein levels in the sciatic nerve explants from 5 days post injury (dpi) to 8 dpi (Figure 1B,C). To localize the expression of Neuritin, we performed immunofluorescent staining on teased sciatic nerve fibres. Briefly, after cultured for 5 or 8 days, nerve fibres were stained with fluorescein phalloidin and Neuritin. Results showed that Neuritin was potently enriched in perinuclear cytoplasm, Schmidt‐Lanterman incisures, inner mesaxons, outer mesaxons at 0 dpi. However, the immunoreactivities of Neuritin were mainly diffused mostly around the myelin ovoids, which are the typical structure of a fragmented myelin sheath (Figure 1C,D). These findings indicate that Neuritn is meaningful for WD after peripheral nerve injury.

### 3.2 Effects of Neuritin on Schwann cells demyelination and fragmentation

**FIGURE 2:**
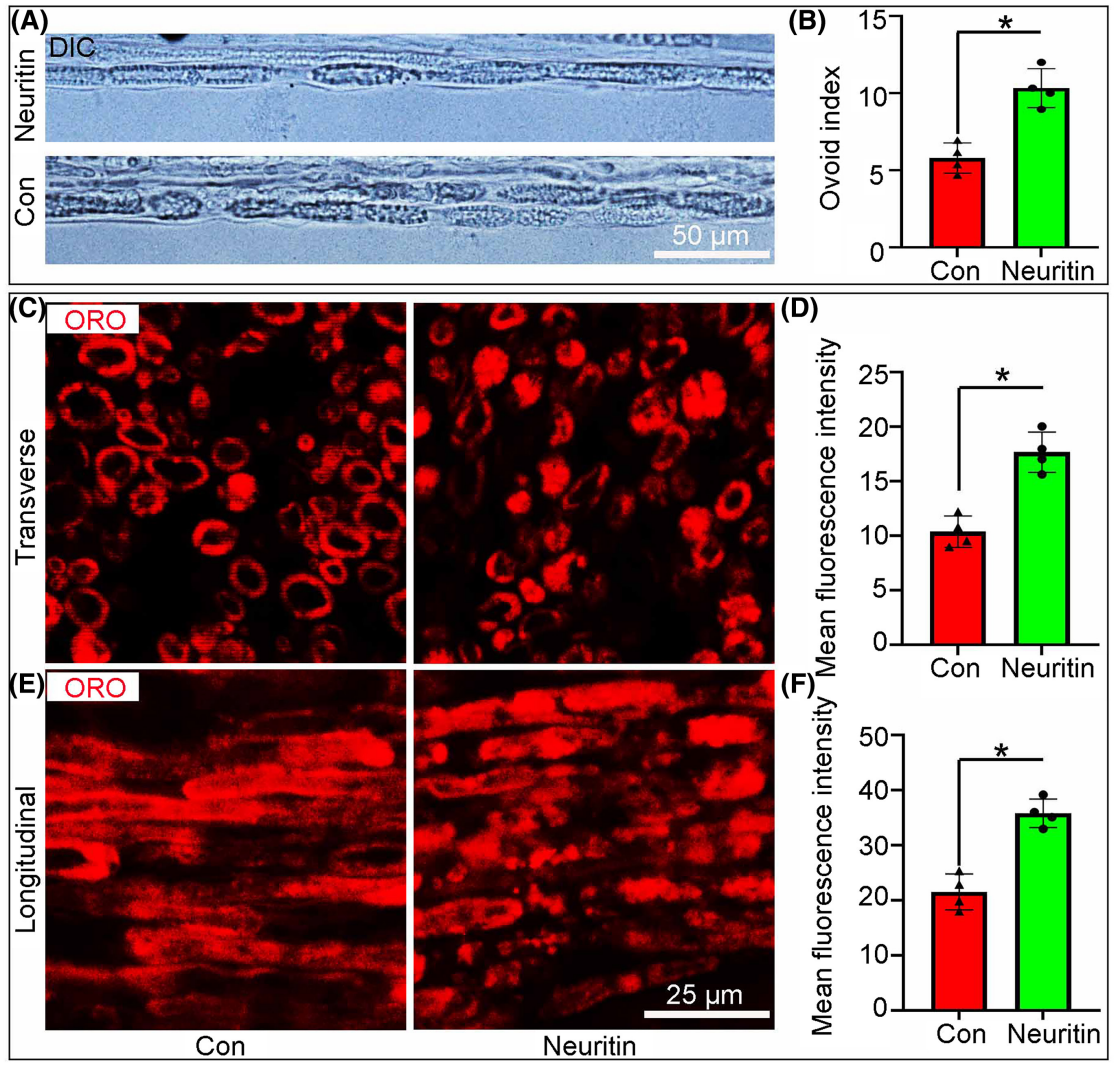
Neuritin promotes myelin degeneration in nerve explants. (A and B) Teased fibres from sciatic nerve explants treated with Neuritin showing more myelin ovoids compared with those of Con group. (C–F) ORO staining showed the myelin sheath degeneration (shrunken myelin with high fluorescence intensity) appeared in nerve explants. And statistical analysis illustrating that the levels of myelin degeneration are signifcantly higher in Neuritin group than those of the Con group. (*n* = 4, *p < 0.05).

First, the sciatic nerve explants cultured for 5 days were teased into single fibres facilitate visualization of the myelin sheath by phase contrast microscopy (Figure 2A). As myelin ovoids was easily identified and quantified, we found that the number of them present in every 200‐μm length of teased fibre (the ovoid index) was highest in the Neuritin group than that in the control group (Figure 2B). ORO staining is used to label lipid droplets in adipocytes.^16^, ^17^ Here, we found that the mean intensity of ORO (an index of degenerated myelin) in the Neuritin group was signifcantly higher than that in the control group (Figure 2C–F).

### 3.3 Effects of Neuritin on axon and myelin degeneration

**FIGURE 3:**
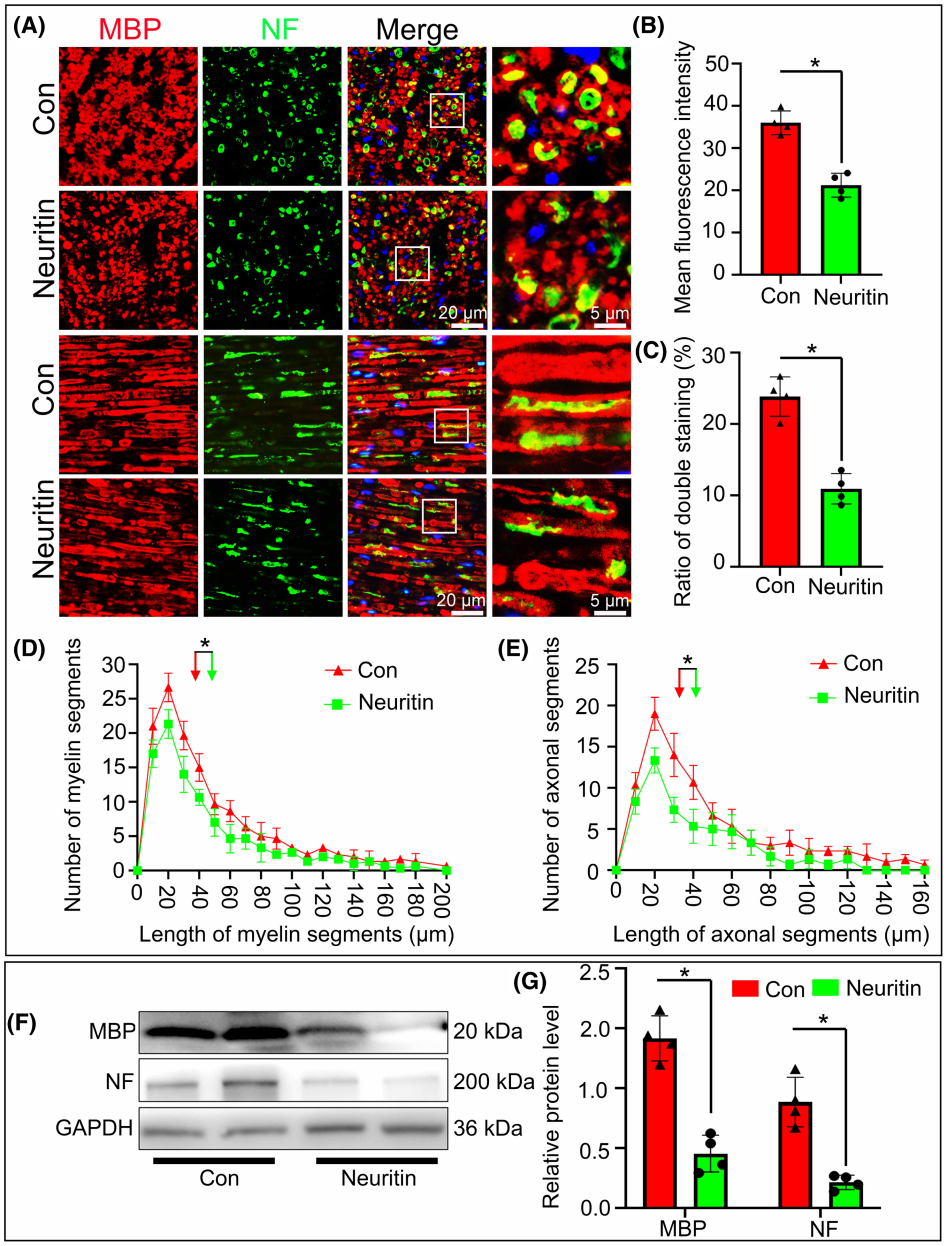
Neuritin promotes axon and myelin degeneration in nerve explants. (A) Immunohistochemistry images showing fragmented axons and myelin sheaths in transverse and longitudinal sections of sciatic nerve explants treated with Neuritin at 5 dpi. (B and C) Mean fluorescence intensity and ratio of double staining of axons and myelin fragments in transverse sections showed that Neuritin could more dramaticly accelerate degeneration compared with Con group. (D and E) Quantifcation of the length distributions of axons and myelin fragments showing both the length of axonal fragments and myelin fragments are signifcantly shorter in the Neuritin group than those of the Con group. (F and G) Western blots and statistical analysis illustrating that the levels of MBP and NF protein are signifcantly lower in Neuritin group than those of the Con group.(*n* = 4, *p < 0.05).

Next, we investigated the role of Neurtin in axonal and myelin degeneration in the explant model.^18^ Immunofuorescence staining on the cross and longitudinal sections showed that the NF staining axons and MBP staining myelin were signifcantly decreased in Neuritin group compared with the control group (Figure 3A–E). Similar trends were observed when NF and MBP levels were assessed by western blot (Figure 3F,G).

### 3.4 Effects of Neuritin on the Schwann cells capacities of phagocytosis and neurotrophins expression

**FIGURE 4:**
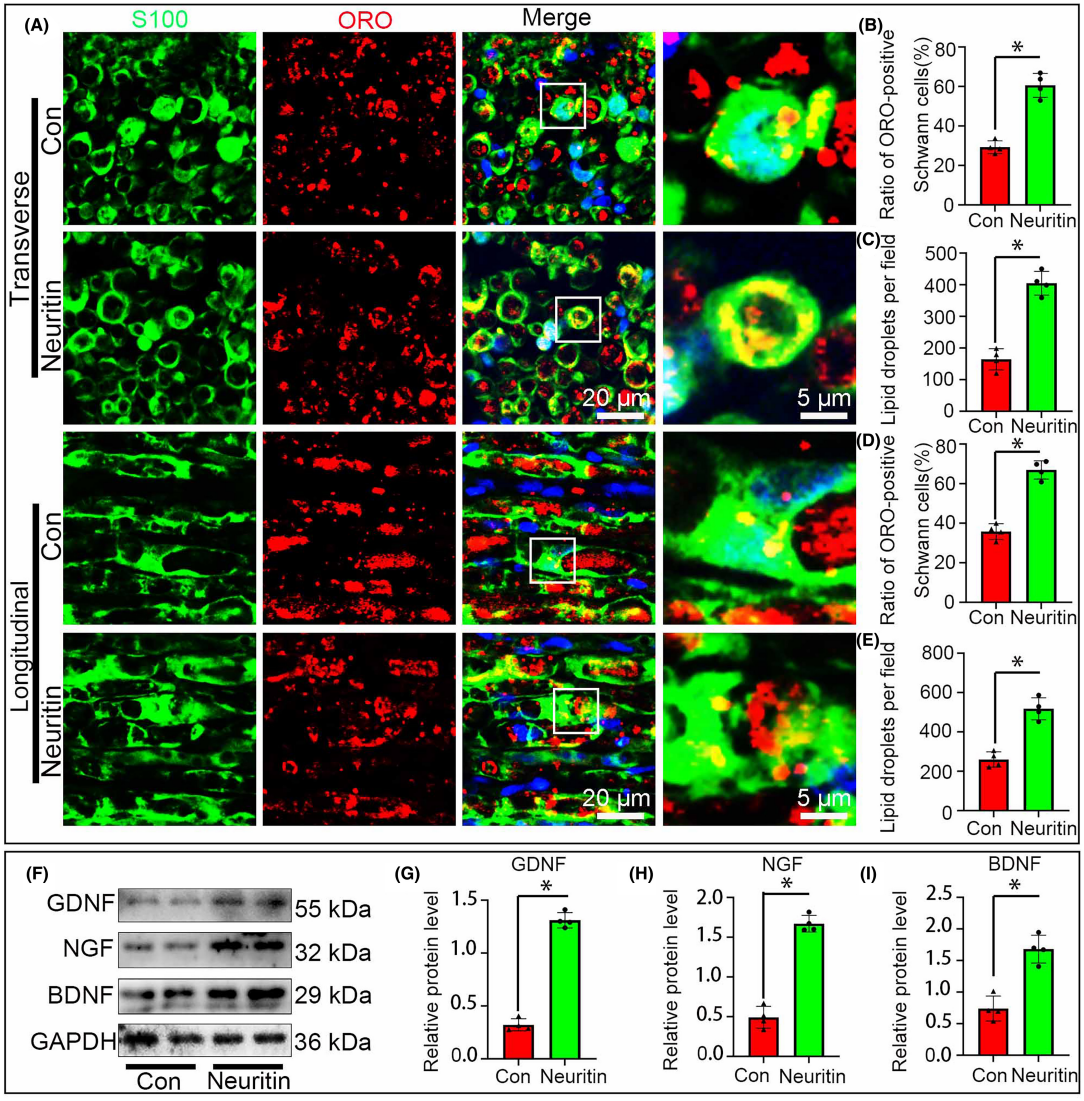
Neuritin facilitates Schwann cells capabilities of phagocytosis and secretion. (A–E) S100 and Lum double staining and quantitative analysis after the Schwann cells cultured with lum at 5 dpi in vitro illustrated that phagocytosis of Schwann cells were significantly enhanced in the Neuritin group compared with the Con group. (F–I) Western blots and statistical analysis illustrated that the levels of GDNF, NGF, BDNF protein are signifcantly higher in Neuritin group than those of the Con group. (*n* = 4, *p < 0.05).

Recent studies indicated that Schwann cells response to debris clearance, namely phagocytosis. Current findings hinted that both phagocytosis and secretion function could be enhanced by Neuritin in Schwann cells. Both cross and longitudinal sections of the nerve explants showed that the ratio of ORO/S100 double positive Schwann cells and the number of ORO+ myelin debris ingested by each S100+ Schwann cell were significantly higher in the Neuritin group than those of the control group (Figure 4A–E). Subsequently, western blotting was used to assess the main neurotrophic factors secreted by Schwann cells, such as GDNF, NGF and BDNF. Current results illustrated that the protein levels of the three neurotrophins were all significantly higher in the Neuritin group compared with the control group (Figure 4F–I) which implies that Neuritin promote neurotrophins expression during WD and may play an vital role in the following nerve regeneration.

### 3.5 Effects of Neuritin on Schwann cell dedifferentiation

**FIGURE 5:**
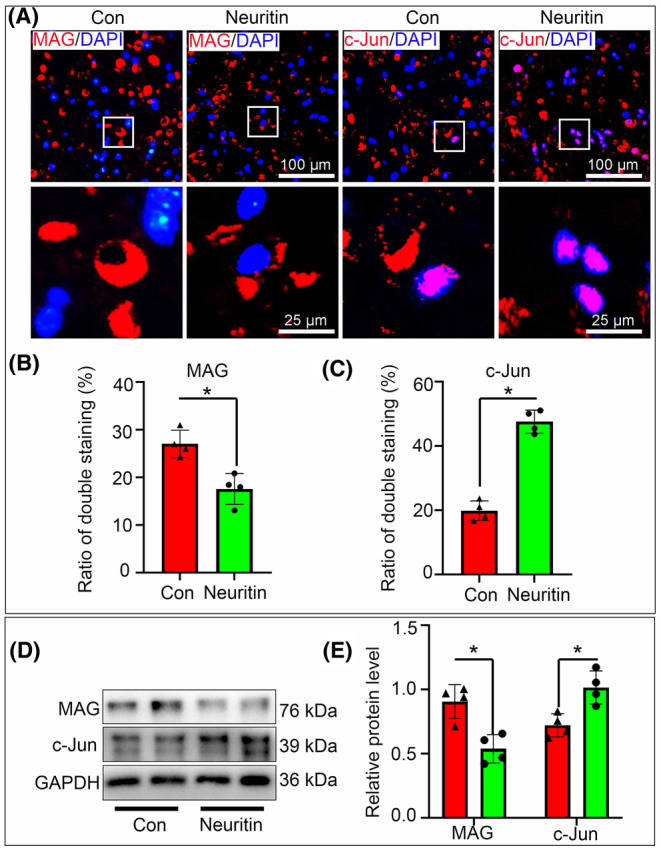
Neuritin facilitates Schwann cell dedifferentiation. (A–C) Immunofluorescence and quantification showed that the number of MAG‐positive cells was significantly lower in the Neuritin group than in Con group. In contrast, the number of c‐Jun‐positive cells in the Neuritin group were remarkably higher than those in Con group. (D and E) Western blots and quantification showed the protein levels of MAG are signifcantly lower in Neuritin group than those of the Con group, while c‐Jun was obviously increased in the Neuritin group.(*n* = 4, *p < 0.05).

As described previously, Schwann cell dedifferentiation and transformation into repair phenotype after nerve injury is a prerequisite for their capabilities of debris clearance and neurotrophins secretion.^19^, ^20^ Herein, c‐Jun (a marker of immature Schwann cells) and MAG (a marker of mature Schwann cells) antibodies were used to identify the role of Neuritin in Schwann cell dedifferentiation via immunostaining and western blotting. Interestingly, the c‐Jun‐positive fluorescent intensity and the number of c‐Jun‐positive cells in the Neuritin group were remarkably higher than those in the control group. In contrast, the number of MAG‐positive cells was significantly lower in the Neuritin group than in the control group (Figure 5A–C). Similarly, the c‐Jun expression was drastically upregulated in the Neuritin group, while MAG was obviously decreased (Figure 5D,E). These data vividly indicated that Neuritin facilitates Schwann cell dedifferentiation in the injured nerve.

### 3.6 Neuritin promotes Wallerian degeneration through PI3K/Akt/mTOR Pathway

**FIGURE 6:**
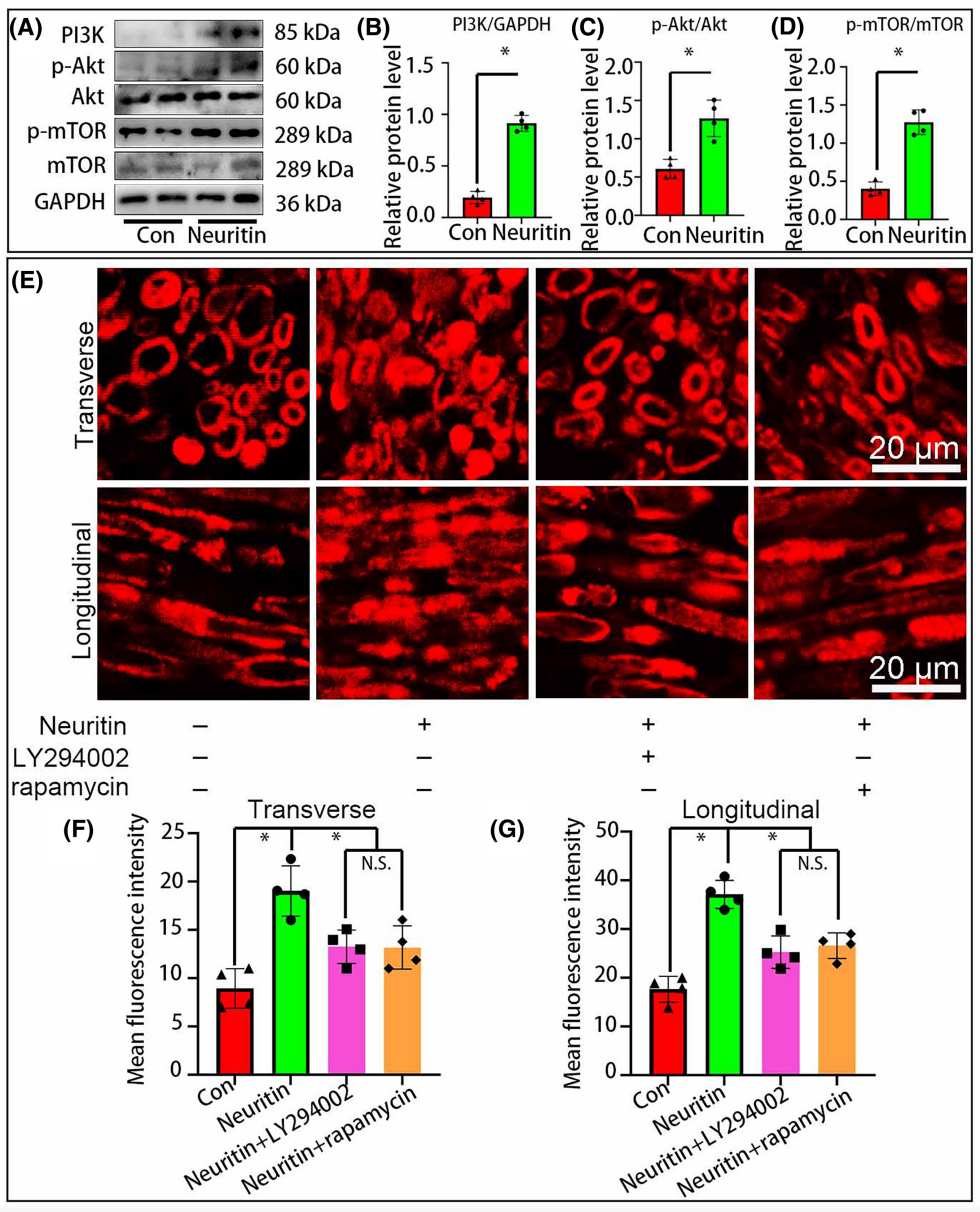
Neuritin activates the PI3K/Akt/mTOR signalling pathway after peripheral nerve injury. (A) Western blots assay was performed in the distal trunk of the injured sciatic nerves and indicated the expression level of PI3K/Akt/mTOR. (B–D) Statistical analysis illustrated that the protein levels of PI3K, p‐Akt/AKT, p‐mTOR/mTOR are signifcantly higher in Neuritin group than those of the Con group. (E–G) ORO staining showed that Neuritin promotes SCs demyelination but inhibited by PI3K inhibitor LY294002 or mTOR inhibitor rapamycin.(*n* = 4, *p < 0.05).

Neuritin is well known for its importance in neurons by regulating the neuronal excitability through PI3K/Akt/mTOR pathway.^21^, ^22^ However, whether Neuritin accelerates the process of WD through PI3K/Akt/mTOR pathway is unknown yet. To test the hypothesis, we compared the expression levels of PI3K/Akt/mTOR by western blotting. As expected, PI3K/Akt/mTOR were dramatically increased in Neuritin group compared to the control group (Figure 6A–D). Next, mTOR inhibitor rapamycin and PI3K inhibitor LY294002 were individually used to assess their effects on the Schwann cells demyelination. As a result, we found that both rapamycin and LY294002 impeded the neuritin induced accelerated demyelination (Figure 6E–G). Above data indicate that Neuritin can up‐regulate the expression of PI3K/Akt/mTOR, which results in promoting the process of WD.

## 4 DISCUSSION

WD, an essential reaction response to nerve injury, is very important for subsequent nerve repair and regeneration.^6^, ^23^ Until now, many critical factors (e.g. Schwann cells demyelination, dedifferentiation and phagocytosis) for WD have been identifed.^24^, ^25^ Schwann cells undergoes a particular plasticity response and is crucial for the successful regeneration of the peripheral nervous systerm. Neuritin is upregulated in hippocampal and cortical neurons in vivo and in vitro by the neurotrophin brain‐derived neurotrophic factor^20^ and induces neurite outgrowth. Although the role of Neuritin as key regulator for neuron regeneration after PNI is well investigated, whether Neuritin also plays a role in Schwann cells during WD remains unclear. In the present study, we found that Neuritin expression was upregulated at 5 or 8 dpi after sciatic nerve injury. Neuritin is predominately located in the perinuclear cytoplasm, Schmidt‐Lanterman incisures, inner mesaxons, outer mesaxons in the normal peripheral nerve fibres. From 5 to 8 dpi, Neuritin is mainly diffused around the myelin ovoids. Since previous studies demonstrated that the first 7 days post nerve injury is a critical period for WD,^26^ therefore, our data suggest the high expression of Neuritin in this critical period might be meaningful for Schwann cell demyelination and the following WD.

In order to figure out this issue, we used a simplified ex vivo WD model of sciatic nerve explants to test the effects of Neuritin on the WD as well as the Schwann cell dedifferentiation. In this model, not any nerve regeneration will occur to interrupt the evaluation of the NF, MAG, and MBP expression which are selected to reflect the levels of axons and myelin, respectively, since axonal regeneration and remyelination might occur before WD is completed.^27^ Moreover, circulating macrophages cannot be recruited into the nerve explants to accelerate WD, so the differences among each group are mainly attributed to the effects of Neuritin on Schwann cells.

In this model, the collected results easily drive us to believe that Neuritin plays a positive role in WD since Neuritin significantly increased the proportion of degenerated myelin and decreased expression levels of NF and MBP, all of which meant the process of WD was accelerated. Meanwhile, immunostaining and western blotting with antibodies to MAG (for differentiated Schwann cells) and c‐Jun (for dedifferentiated Schwann cells) indicated Neuritin enhanced the Schwann cell dedifferentiation. WD occurs at the early stage after nerve injury, which can create a favourable microenvironment for the following nerve regeneration. The dedifferentiated Schwann cell play a key role in debris clearance to accelerate WD, and also can produce neurotrophins by the dozens to promote nerve regeneration.^28^, ^29^, ^30^ It is well known that the PI3K/Akt/mTOR signalling pathway is essential for Schwann cells autophagy, migration and myelination.^31^, ^32^, ^33^ Further, recently study founded that Neuritin suppresses oesophageal cancer growth and up‐regulates Kv4.2‐mediated transient outward K^+^ current through the PI3K/Akt/mTOR pathway in rat cerebellar granule neurons.^11^ In order to explore whether Neuritin plays a role in the Schwann cell demyelination during WD is caused by regulating PI3K/Akt/mTOR pathway, we set up the ex vivo nerve explants model and found that an up‐regulation of PI3K, p‐Akt, p‐mTOR was revealed by western blotting in Neuritin group. Finally, we used mTOR inhibitor rapamycin and PI3K inhibitor LY294002 were individually used to assess their effects on the Schwann cells demyelination, by which the effect of Neuritin of the Schwann cell demyeliation and WD was efficiently reversed.

In conclusion, present data indicate that Neuritin plays a positive role in WD by up‐regulating the PI3K/Akt/mTOR signalling pathway. Thus, our results provide a new insight into Neuritin regulation of peripheral nerve degeneration and suggest a potential therapeutic target for recovery of peripheral nerve injury.

## AUTHOR CONTRIBUTIONS

**Jingmin Liu:** Formal analysis (lead); funding acquisition (supporting); methodology (lead); supervision (equal); visualization (lead); writing – original draft (equal); writing – review and editing (lead). **Xin Guan:** Data curation (equal); formal analysis (equal); software (equal); writing – original draft (equal). **Shuai Zheng:** Methodology (equal); resources (equal). **Jiawei Shi:** Formal analysis (equal); software (equal). **Xiaobo Wang:** Data curation (equal); investigation (equal). **Zetao Shen:** Data curation (equal). **Zefu Chen:** Data curation (equal). **Congrui Liao:** Formal analysis (equal). **Zhongmin Zhang:** Funding acquisition (supporting); supervision (equal); writing – review and editing (equal).

## FUNDING INFORMATION

This study was granted by the Presidential Foundation of NanFang Hospital (2023A039); National key R & D project (SQ2022YFC2500092).

## CONFLICT OF INTEREST STATEMENT

The authors declare no competing interests.

## Data Availability

The data that support the findings of this study are available from the corresponding author upon reasonable request.

## References

[1] Zhao QR , Lu JM , Li ZY , Mei YA . Neuritin promotes neurite and spine growth in rat cerebellar granule cells via L‐type calcium channel‐mediated calcium influx. J Neurochem. 2018;147(1):40‐57. doi:10.1111/jnc.14535 29920676 PMC6220818

[2] Naeve GS , Ramakrishnan M , Kramer R , Hevroni D , Citri Y , Theill LE . Neuritin: a gene induced by neural activity and neurotrophins that promotes neuritogenesis. Proc Natl Acad Sci USA. 1997;94(6):2648‐2653. doi:10.1073/pnas.94.6.2648 9122250 PMC20143

[3] Karamoysoyli E , Burnand RC , Tomlinson DR , Gardiner NJ . Neuritin mediates nerve growth factor‐induced axonal regeneration and is deficient in experimental diabetic neuropathy. Diabetes. 2008;57(1):181‐189. doi:10.2337/db07-0895 17909094

[4] Xi C , Zhang Y , Yan M , et al. Exogenous neuritin treatment improves survivability and functions of Schwann cells with improved outgrowth of neurons in rat diabetic neuropathy. J Cell Mol Med. 2020;24(17):10166‐10176. doi:10.1111/jcmm.15627 32667138 PMC7520300

[5] Huang T , Li H , Zhang S , Liu F , Wang D , Xu J . Nrn1 overexpression attenuates retinal ganglion cell apoptosis, promotes axonal regeneration, and improves visual function following optic nerve crush in rats. J Mol Neurosci. 2021;71(1):66‐79. doi:10.1007/s12031-020-01627-3 32607759

[6] Li X , Zhang T , Li C , et al. Electrical stimulation accelerates Wallerian degeneration and promotes nerve regeneration after sciatic nerve injury. Glia. 2023;71(3):758‐774. doi:10.1002/glia.24309 36484493

[7] Liu J , Li L , Zou Y , et al. Role of microtubule dynamics in Wallerian degeneration and nerve regeneration after peripheral nerve injury. Neural Regen Res. 2022;17(3):673‐681. doi:10.4103/1673-5374.320997 34380909 PMC8504388

[8] Zou Y , Zhang J , Liu J , et al. SIRT6 negatively regulates Schwann cells dedifferentiation via targeting c‐Jun during Wallerian degeneration after peripheral nerve injury. Mol Neurobiol. 2022;59(1):429‐444. doi:10.1007/s12035-021-02607-3 34708329

[9] Xu J , Wen J , Fu L , et al. Macrophage‐specific RhoA knockout delays Wallerian degeneration after peripheral nerve injury in mice. J Neuroinflammation. 2021;18(1):234. doi:10.1186/s12974-021-02292-y 34654444 PMC8520251

[10] Lee SH , Kim NS , Choi M , et al. LGI1 governs Neuritin‐mediated resilience to chronic stress. Neurobiol Stress. 2021;15:100373. doi:10.1016/j.ynstr.2021.100373 34401409 PMC8350063

[11] Yao JJ , Gao XF , Chow CW , Zhan XQ , Hu CL , Mei YA . Neuritin activates insulin receptor pathway to up‐regulate Kv4.2‐mediated transient outward K^+^ current in rat cerebellar granule neurons. J Biol Chem. 2012;287(49):41534‐41545. doi:10.1074/jbc.M112.390260 23066017 PMC3510849

[12] Guo J , Wang L , Zhang Y , et al. Abnormal junctions and permeability of myelin in PMP22‐deficient nerves. Ann Neurol. 2014;75(2):255‐265. doi:10.1002/ana.24086 24339129 PMC4206215

[13] Jung J , Cai W , Lee HK , et al. Actin polymerization is essential for myelin sheath fragmentation during Wallerian degeneration. J Neurosci. 2011;31(6):2009‐2015. doi:10.1523/JNEUROSCI.4537-10.2011 21307239 PMC3071261

[14] Zou Y , Wu S , Wen F , Ge Y , Luo S . PGC‐1alpha inhibits Schwann cell dedifferentiation and delays peripheral nerve degeneration by targeting PON1. Cell Mol Neurobiol. 2023;43(7):3767‐3781. doi:10.1007/s10571-023-01395-9 37526811 PMC11409949

[15] Liu J , Ma X , Hu X , et al. Schwann cell‐specific RhoA knockout accelerates peripheral nerve regeneration via promoting Schwann cell dedifferentiation. Glia. 2023;71(7):1715‐1728. doi:10.1002/glia.24365 36971019

[16] Schachner‐Nedherer AL , Werzer O , Kornmueller K , Prassl R , Zimmer A . Biological activity of miRNA‐27a using peptide‐based drug delivery systems. Int J Nanomedicine. 2019;14(14):7795‐7808. doi:10.2147/IJN.S208446 31576124 PMC6768125

[17] Velickovic K , Lugo LH , Surrati A , et al. Targeting glutamine synthesis inhibits stem cell adipogenesis in vitro. Cell Physiol Biochem. 2020;54(5):917‐927. doi:10.33594/000000278 32946687

[18] Li L , Xu Y , Wang X , et al. Ascorbic acid accelerates Wallerian degeneration after peripheral nerve injury. Neural Regen Res. 2021;16(6):1078‐1085. doi:10.4103/1673-5374.300459 33269753 PMC8224114

[19] Balakrishnan A , Belfiore L , Chu TH , et al. Insights into the role and potential of Schwann cells for peripheral nerve repair from studies of development and injury. Front Mol Neurosci. 2020;13:608442. doi:10.3389/fnmol.2020.608442 33568974 PMC7868393

[20] Su Q , Nasser MI , He J , et al. Engineered Schwann cell‐based therapies for injury peripheral nerve reconstruction. Front Cell Neurosci. 2022;16:865266. doi:10.3389/fncel.2022.865266 35602558 PMC9120533

[21] Du W , Gao A , Herman J , et al. Methylation of NRN1 is a novel synthetic lethal marker of PI3K‐Akt‐mTOR and ATR inhibitors in esophageal cancer. Cancer Sci. 2021;112(7):2870‐2883. doi:10.1111/cas.14917 33931924 PMC8253287

[22] Yao JJ , Zhao QR , Liu DD , Chow CW , Mei YA . Neuritin up‐regulates Kv4.2 alpha‐subunit of potassium channel expression and affects neuronal excitability by regulating the calcium‐calcineurin‐NFATc4 signaling pathway. J Biol Chem. 2016;291(33):17369‐17381. doi:10.1074/jbc.M115.708883 27307045 PMC5016134

[23] Cheng Q , Wang YX , Yu J , Yi S . Critical signaling pathways during Wallerian degeneration of peripheral nerve. Neural Regen Res. 2017;12(6):995‐1002. doi:10.4103/1673-5374.208596 28761435 PMC5514877

[24] Park HT , Kim JK , Tricaud N . The conceptual introduction of the "demyelinating Schwann cell" in peripheral demyelinating neuropathies. Glia. 2019;67(4):571‐581. doi:10.1002/glia.23509 30378179

[25] Jang SY , Shin YK , Park SY , et al. Autophagic myelin destruction by Schwann cells during Wallerian degeneration and segmental demyelination. Glia. 2016;64(5):730‐742. doi:10.1002/glia.22957 26712109

[26] Jia B , Huang W , Wang Y , et al. Nogo‐C inhibits peripheral nerve regeneration by regulating Schwann cell apoptosis and dedifferentiation. Front Neurosci. 2020;14:616258. doi:10.3389/fnins.2020.616258 33584179 PMC7873940

[27] Elsayed H , Faroni A , Ashraf MR , et al. Development and characterisation of an in vitro model of Wallerian degeneration. Front Bioeng Biotechnol. 2020;8:784. doi:10.3389/fbioe.2020.00784 32754584 PMC7365951

[28] Jessen KR , Mirsky R . The repair Schwann cell and its function in regenerating nerves. J Physiol‐London. 2016;594(13):3521‐3531. doi:10.1113/JP270874 26864683 PMC4929314

[29] Pandey S , Mudgal J . A review on the role of endogenous neurotrophins and Schwann cells in axonal regeneration. J Neuroimmune Pharmacol. 2022;17(3–4):398‐408. doi:10.1007/s11481-021-10034-3 34843075 PMC9810669

[30] Gao D , Huang Y , Sun X , Yang J , Chen J , He J . Overexpression of c‐Jun inhibits erastin‐induced ferroptosis in Schwann cells and promotes repair of facial nerve function. J Cell Mol Med. 2022;26(8):2191‐2204. doi:10.1111/jcmm.17241 35191156 PMC8995448

[31] Ishii A , Furusho M , Bansal R . Mek/ERK1/2‐MAPK and PI3K/Akt/mTOR signaling plays both independent and cooperative roles in Schwann cell differentiation, myelination and dysmyelination. Glia. 2021;69(10):2429‐2446. doi:10.1002/glia.24049 34157170 PMC8373720

[32] Yan JN , Zhang HY , Li JR , et al. Schwann cells differentiated from skin‐derived precursors provide neuroprotection via autophagy inhibition in a cellular model of Parkinson's disease. Neural Regen Res. 2022;17(6):1357‐1363. doi:10.4103/1673-5374.327353 34782582 PMC8643066

[33] Gao D , Tang T , Zhu J , Tang Y , Sun H , Li S . CXCL12 has therapeutic value in facial nerve injury and promotes Schwann cells autophagy and migration via PI3K‐AKT‐mTOR signal pathway. Int J Biol Macromol. 2019;124:460‐468. doi:10.1016/j.ijbiomac.2018.10.212 30391592

